# Clinical significance of the sub-classification of 71 cases mucinous breast carcinoma

**DOI:** 10.1186/2193-1801-2-481

**Published:** 2013-09-23

**Authors:** Shinichiro Kashiwagi, Naoyoshi Onoda, Yuka Asano, Satoru Noda, Hidemi Kawajiri, Tsutomu Takashima, Masahiko Ohsawa, Seiichi Kitagawa, Kosei Hirakawa

**Affiliations:** Department of Surgical Oncology, Osaka City University Graduate School of Medicine, 1-4-3 Asahi-machi, Abeno-ku, Osaka, Japan; Department of Diagnostic Pathology, Osaka City University Graduate School of Medicine, 1-4-3 Asahi-machi, Abeno-ku, Osaka, Japan; Department of Physiology, Osaka City University Graduate School of Medicine, 1-4-3 Asahi-machi, Abeno-ku, Osaka, Japan

**Keywords:** Mucinous breast carcinoma, Subclassification, Breast cancer, Hypocellular variant, Hypercellular variant

## Abstract

**Objective:**

Mucinous breast carcinoma (MBC) is classified into mixed mucinous breast carcinoma (MMBC) and pure mucinous breast carcinoma (PMBC) based on whether the tumor is with or without a component of invasive ductal carcinoma, respectively. PMBC is subtyped into hypocellular PMBC (PMBC-A) and hypercellular PMBC (PMBC-B).

**Methods:**

Of 1,760 primary breast carcinomas, 71 were diagnosed as MBC, and were subtyped for comparison purposes.

**Results:**

Seventy-one of all breast cancers (4.0%) were MBC, and consisted of 23 MMBC, 32 PMBC-A and 16 PMBC-B. The MBC tumors were more often hormone receptor-positive and HER2-negative than non-MBC tumors. Patients with MMBC, PMBC-B or PMBC-A, in this order, had significantly higher recurrence rates than non-MBC cases (p=0.006, log-rank).

**Conclusions:**

In the NCCN guidelines, MBC is also regarded as “a histological type with a favorable prognosis” in a uniform manner, and “treatment for a histological type with a favorable prognosis” is recommended. However, the results of this study suggest that sub-classification-based, individualized therapeutic strategies should be considered.

## Introduction

Mucinous breast carcinoma (MBC), a special type of breast carcinoma, is the second most common cancer after lobular carcinoma, accounting for 1.3 - 5.4% of all breast cancers (Ellis et al. 2003; Fentiman et al. 1977; Komaki et al. 1988; Scopsi et al. 1994; Anan et al. 2001). According to the current General Rules for Clinical and Pathological Recording of Breast Cancer, MBC is characterized by the fact that the cancer cells produce mucus, and nests of mucinous cancer cells occupy almost the entire tumor mass (Wilson et al. 1955). In the WHO classification of breast tumors, MBC is described as a subtype of mucinous carcinoma and other tumors with abundant mucus (mucin-producing carcinomas) (Ellis et al. 2003). It is histologically subtyped into mixed mucinous breast carcinoma (MMBC) containing a component of conventional invasive ductal carcinoma, and pure mucinous breast carcinoma (PMBC), in which nests of mucinous cancer cells occupy almost the entire tumor mass, and which contains no component of conventional invasive ductal carcinoma (Capella et al. 1980; Ranade et al. 2010; Rosen 2008; André et al. 1995). PMBC may be subtyped into a hypocellular variant (PMBC-A) showing a tubular, cribriform, cord-like, micropapillary or papillary growth pattern, and a hypercellular variant (PMBC-B) growing in solid nests (Capella et al. 1980; Ranade et al. 2010). In this study, we clinicopathologically analyzed MBC according to these subtypes.

## Materials and methods

### Patient background

A total of 1,760 patients with primary breast cancer underwent surgery between January 2000 and December 2012. Of these patients, 71 (4.0%) were diagnosed with MBC. These MBC cases were subtyped into MMBC, PMBC-A and PMBC-B for comparison purposes. The median follow-up period was 7.8 years (range, 0.5 – 10.1 years).

### Immunohistochemistry

The hormone receptor (HR), human epidermal growth factor receptor 2 (HER2) expression and proliferative activity, as measured by the Ki67 labeling index, were evaluated by immunohistochemical staining of formalin-fixed, paraffin-embedded surgical breast cancer specimens using the ABC method. For antigen activation, deparaffinized sections were immersed in 0.01 M citrate buffer (pH 6.0), and heated at 105°C for 10 min. Primary antibodies against the ER (clone 1D5, dilution 1:80; Dako, Carpinteria, California, USA), PR (clone PgR636, dilution 1:100; Dako), HER2 (Hercep Test™, Dako) and Ki67 (clone MIB-1, dilution 1:100; Dako) were used. Endogenous peroxidase activity was blocked with 0.3% H_2_O_2_ in methanol, color was developed with DAB (3, 3-diaminobenzidine, tetrahydrochloride) and the sections were counterstained with Meyer’s hematoxylin. A pathological diagnosis was made by two pathologists who specialized in mammary gland pathology using the blind method to confirm the objectivity and reproducibility of the diagnosis.

### Immunohistochemical scoring

For the hormone receptors, any staining was considered to indicate a positive sample. HER2 reactivity was assessed according to the ASCO/CAP guidelines. The Ki67 labeling index was evaluated as a percentage of positive cells among more than 1,000 cancer cells in five high-powered fields (HPFs) of the deepest portion and margin of the superficial layer of the tumor, and a percentage of 14% or higher was considered to be positive.

### Statistical analysis

Statistical analysis was performed using SPSS version 13.0 statistical software (IBM, Armonk, New York, USA). Clinicopathological factors were analyzed in MBC patients using the chi-square test. Disease-free survival rates were calculated using the Kaplan-Meier method, and significance was assessed using the log-rank test. A value of p < 0.05 was considered to be significant.

## Results

Of the 1,760 patients who underwent surgery for primary breast cancer, 71 (4.0%), 23 (32.4%), 32 (45.1%) and 16 (22.5%) had MBC, MMB, PMBC-A and PMBC-B, respectively. Table 1 shows their clinicopathological characteristics. Their mean age was 61.8 years (range, 26–95 years; median, 64 years). The mean tumor diameter was 3.1 cm (range, 0.5–12.3 cm; median, 2.5 cm). Eight (11.3%), fifteen (21.1%), and 31 patients (43.7%) had lymph node metastasis, vascular invasion and an extensive intraductal component (EIC), respectively. Breast cancer subtyping based on the hormone receptor (HR) and HER2 expression showed that, of the 71 tumors, 68 (95.8%), one (1.4%) and two (2.8%) were HR-positive/HER2-negative, HR-negative/HER2-postive and HR-negative/HER2-negative (so-called triple-negative), respectively. Of the seven patients with recurrence, six (85.7%) had MMBC, none had PMBC-A, one (14.3%) had PMBC-B, and none of the patients had died.

**Table 1:** **Demographical data of 71 patients with mucinous carcinoma of the breast**

Parameters(n=71)	Percentage
Age	mean 61.8 (26 – 95, median 64)
Tumor diameter (cm)	mean 3.1 (0.5 – 12.3, median 2.5)
Tumor location	39 (54.9%)/32 (45.1%)
right/left	
Subtype	23 (32.4%)/32 (45.1%)/16 (22.5%)
MMBC/PMBC-A / PMBC-B	
Pathological stage	23 (32.4%)/40 (56.3%)/8 (11.3%)
I/II/III	
pT	23 (32.4%)/38 (53.5%)/6 (8.5%)/ 4 (5.6%)
T1/T2/T3/T4	
pLymph node status	8 (11.3%)/63 (88.7%)
Positive/Negative	
Lymphovascular invasion	15 (21.1%)/56 (78.9%)
Positive/Negative	
Nuclear grade	55 (77.5%)/15 (21.1%)/1 (1.4%)
1/2/3	
EIC	31 (43.7%)/40 (56.3%)
Positive/Negative	
Hormone receptor status	68 (95.8%)/3 (4.2%)
Positive/Negative	
HER 2 status	1 (1.4%)/70 (98.6%)
Positive/Negative	
Ki67	8 (11.3%)/63 (88.7%)
Positive/Negative	
Recurrence	7 (9.9%)/64 (90.1%)
Recurrence/no- Recurrence	
Recurrence cases (n=7)	6 (85.7%)/0 (0.0%)/1 (14.3%)
MMBC/PMBC-A/PMBC-B	

*MMBC* mixed mucinous carcinoma, *PMBC* pure mucinous carcinoma, *EIC* extensive intraductal component.

The clinicopathological analysis revealed that MBCs had a significantly larger diameter than non-MBCs (p=0.001), and were significantly more frequently HR-positive (p<0.001) and negative for HER2 overexpression (p<0.001) (Table 2).

**Table 2:** **Clinicopathologic feature of 71 mucinous breast cancers in 1760 breast carcinomas**

Parameters	MBC (n=71)	Non-MBC(n=1689)	p value
Age at operation	38 (53.5%)	965 (57.1%)	0.547
≤64	33 (46.5%)	724 (42.9%)	
>64			
Pathological stage	23 (32.4%)	799 (47.3%)	0.014
I	48 (67.6%)	890 (52.7%)	
II and III			
pTumor size	25 (35.2%)	937 (55.5%)	0.001
≤2 cm	46 (64.8%)	752 (45.5%)	
>2 cm			
pLymph node status	63 (88.7%)	1270 (75.2%)	0.009
Negative	8 (11.3%)	419 (24.8%)	
Positive			
Lymphovascular invasion	56 (78.9%)	1276 (75.5%)	0.522
Negative	15 (21.1%)	413 (24.5%)	
Positive			
HR (ER and/or PR)	3 (4.2%)	477 (28.2%)	< 0.001
Negative	68 (95.8%)	1212 (71.8%)	
Positive			
HER 2 status	70 (98.6%)	1310 (77.6%)	< 0.001
Negative	1 (1.4%)	379 (22.4%)	
Positive			

*MBC* mucinous breast carcinoma.

*HR* Hormone receptor.

**Figure 1:**
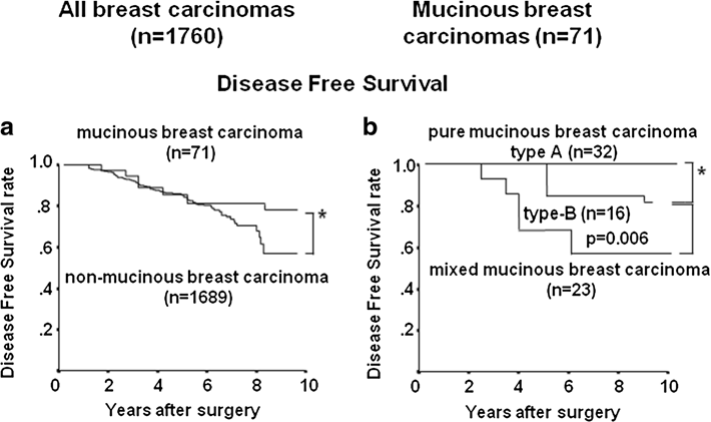
**Correlation between the mucinous carcinoma phenotype and disease**-**free survival in the breast cancer series.** The mixed mucinous breast carcinoma patients experienced significantly poorer outcomes in all breast cancers **(a)** and mucinous carcinomas **(b)**. (NS; not significant).

Among all patients (n=1,760), there was no significant difference in the DFS between those with MBC and those with non-MBC tumors (p=0.614, log-rank test) (Figure 1a). However, a subtype analysis showed that patients with MMBC, PMBC-B or PMBC-A, in this order, had a significantly higher recurrence rate than those with non-MBC tumors (p=0.006, log-rank test) (Figure 1b). Among the 71 MBCs, MMBC showed a significantly higher Ki67 labeling index (P=0.010) and nuclear grade (p <0.001) than PMBC, and these were not correlated with the HR or HER2 expression (Table 3). Among the 48 PMBCs, no significant differences in the clinicopathological characteristics were noted between patients with PBMC-A and those with PMBC-B.

**Table 3:** **Correlations between subtype and clinicopathological parameters in 71 mucinous breast carcinomas**

Parameters	MBC (n=71)		p value	PMBC (n=48)		p value
	MMBC (n=23)	PMBC (n=48)		PMBC-A (n=32)	PMBC-B (n=16)	
Age at operation	14 (60.9%)	24 (50.0%)	0.390	14 (43.8%)	10 (62.5%)	0.220
≤64	9 (39.1%)	24 (50.0%)		18 (56.2%)	6 (37.5%)	
>64						
Pathological stage	9 (39.1%)	14 (29.2%)	0.401	8 (25.0%)	6 (37.5%)	0.369
I	14 (60.9%)	34 (70.8%)		24 (75.0%)	10 (62.5%)	
II and III						
pTumor size	11 (47.8%)	14 (29.2%)	0.123	8 (25.0%)	6 (37.5%)	0.175
≤2 cm	12 (52.2%)	34 (70.8%)		24 (75.0%)	10 (62.5%)	
>2 cm						
pLymph node status	19 (82.6%)	44 (91.7%)	0.162	30 (93.8%)	14 (87.5%)	0.460
Negative	4 (17.4%)	4 (8.3%)		2 (6.2%)	2 (12.5%)	
Positive						
Lymphovascular invasion	16 (69.6%)	40 (83.3%)	0.184	29 (90.6%)	11 (68.8%)	0.057
Negative	7 (30.4%)	8 (16.7%)		3 (9.4%)	5 (32.2%)	
Positive						
HR (ER and / or PR)	1 (4.3%)	2 (4.2%)	0.454	1 (3.1%)	1 (6.3%)	0.453
Negative	22 (95.7%)	46 (95.8%)		31 (96.9%)	15 (93.7%)	
Positive						
HER2 status	23 (100.0%)	47 (97.9%)	0.676	31 (96.9%)	16 (100.0%)	0.667
Negative	0 (0.0%)	1 (2.1%)		1 (3.1%)	0 (0.0%)	
Positive						
Ki67	17 (73.9%)	46 (95.8%)	0.010	30 (93.8%)	16 (100.0%)	0.439
Negative	6 (26.1%)	2 (4.2%)		2 (6.2%)	0 (0.0%)	
Positive						
Nuclear grade	11 (47.8%)	44 (91.7%)	< 0.001	30 (93.8%)	14 (87.5%)	0.306
1	12 (52.2%)	4 (8.3%)		2 (6.2%)	2 (22.5%)	
2 and 3						
EIC	12 (52.2%)	28 (58.3%)	0.624	20 (62.5%)	8 (50.0%)	0.174
Negative	11 (47.8%)	20 (41.7%)		12 (37.5%)	8 (50.0%)	
Positive						

*BMC* breast mucinous carcinoma, *MMBC* mixed mucinous carcinoma, *PMBC* pure mucinous carcinoma, *HR* Hormone receptor, *EIC* extensive intraductal component.

**Figure 2:**
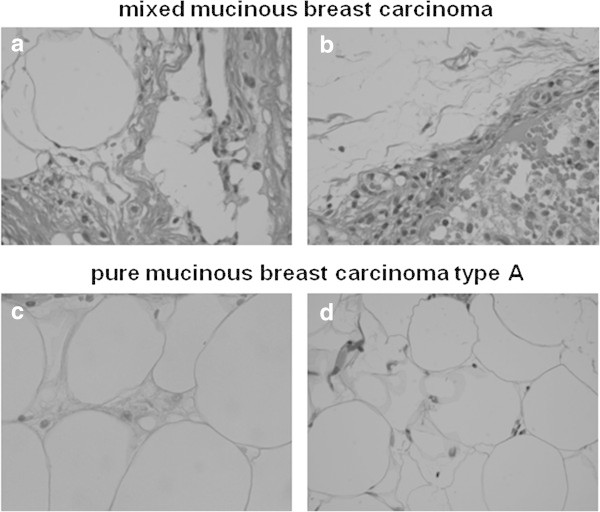
**Histological findings** (**H**.**E**. **stain x 400). (a)** primary lesion of mixed mucinous breast carcinoma. **(b)** metastatic lymph node of mixed mucinous breast carcinoma. **(c)** primary lesion of pure mucinous breast carcinoma type A. **(d)** metastatic lymph node of pure mucinous breast carcinoma type A.

The patients with recurrence were divided into those with and without lymph node metastasis, and their primary tumors and metastatic lymph nodes were pathologically examined. As a result, other pathological types coexisting with MBC (Figure 2a) were also confirmed to be present in the metastatic lymph nodes (Figure 2b). A mucus-rich lesion, as seen in the primary tumor (Figure 2c), was also observed in the metastatic lymph nodes (Figure 2d) of the patients with PMBC-A who had no recurrence in the presence of lymph node metastasis.

## Discussion

It has been reported that MBC shows a favorable prognosis, with a 10-year survival rate of more than 90% (Fentiman et al. 1977; Komaki et al. 1988; Di Saverio et al. 2008). Since a higher ratio of the extracellular mucus component to the cancer cell component is associated with a more favorable prognosis, PMBC-A, PMBC-B and MMBC, in this order, would be expected to show a favorable prognosis (Komaki et al. 1988; Ranade et al. 2010; Clayton 1986). In this study, there was no significant difference in the DFS between the patients with MBC and those with non-MBC tumors. However, a subtype analysis showed that patients with MMBC had a significantly higher recurrence rate than those with PMBC, suggesting the usefulness of breast cancer sub-classification.

Of the seven patients with recurrence in the present study, six (85.7%) had MMBC, none had PMBC-A and one (14.3%) had PMBC-B. Of the eight patients with lymph node metastasis, four (three with MMBC and one with PMBC-B) had recurrence. On the other hand, two patients with PMBC-A had no recurrence in the presence of lymph node metastasis. This suggests that PMBC-A with a high proportion of mucus shows a favorable prognosis even in the presence of lymph node metastasis.

From the viewpoint of the intrinsic subtype, MBC tumors were frequently HR-positive/HER2-negative, and often of the so-called luminal A type (Ki67 labeling index <14%). Many researchers have reported that most MBCs are luminal A type, and that this is a clinicopathological characteristic of MBC (Komaki et al. 1988; Diab et al. 1999; Cao et al. 2012; Shousha et al. 1989). Hormone therapy is a common treatment option used for patients with luminal A-type breast cancer, but some MBCs are resistant to treatment, suggesting that MMBC, which are considered to have a relatively poor prognosis based on the present results, may have different biological properties even within the same tumor type.

Thus, with regard to the therapeutic strategies based on the sub-classification of mucinous carcinoma of the mammary gland, “standard treatment” for a coexisting histological type should be performed in patients with MMBC, which shows a high recurrence rate (NCCN Clinical Practice Guidelines in Oncology Breast Cancer v.2 2012). Among patients with PMBC, “standard treatment” or “treatment for a histological type with a favorable prognosis” should be selected based on the presence or absence of lymph node metastasis in those with PMBC-B. On the other hand, in those with PMBC-A, “treatment for a histological type with a favorable prognosis” may be selected, regardless of the presence or absence of lymph node metastasis.

In the NCCN guidelines, MBC is also regarded as “a histological type with a favorable prognosis” in a uniform manner, and “treatment for a histological type with a favorable prognosis” is recommended. However, the results of this study suggest that sub-classification-based, individualized therapeutic strategies should therefore be considered.
